# Ultrastructural Alterations in Cells of Sunflower Linear Glandular Trichomes during Maturation

**DOI:** 10.3390/plants10081515

**Published:** 2021-07-23

**Authors:** Evelyn Amrehn, Otmar Spring

**Affiliations:** Department of Biochemistry of Plant Secondary Metabolism (190b), Institute of Biology, University of Hohenheim, Garbenstraße 30, 70593 Stuttgart, Germany; Evelyn.Amrehn@hotmail.de

**Keywords:** trichome cytology, glands, *Helianthus annuus*, terpenes, flavonoids

## Abstract

Sunflower and related taxa are known to possess a characteristic type of multicellular uniseriate trichome which produces sesquiterpenes and flavonoids of yet unknown function for this plant. Contrary to the metabolic profile, the cytological development and ultrastructural rearrangements during the biosynthetic activity of the trichome have not been studied in detail so far. Light, fluorescence and transmission electron microscopy were employed to investigate the functional structure of different trichome cells and their subcellular compartmentation in the pre-secretory, secretory and post-secretory phase. It was shown that the trichome was composed of four cell types, forming the trichome basis with a basal and a stalk cell, a variable number (mostly from five to eight) of barrel-shaped glandular cells and the tip consisting of a dome-shaped apical cell. Metabolic activity started at the trichome tip sometimes accompanied by the formation of small subcuticular cavities at the apical cell. Subsequently, metabolic activity progressed downwards in the upper glandular cells. Cells involved in the secretory process showed disintegration of the subcellular compartments and lost vitality in parallel to deposition of fluorescent and brownish metabolites. The subcuticular cavities usually collapsed in the early secretory stage, whereas the colored depositions remained in cells of senescent hairs.

## 1. Introduction

Plant trichomes are highly essential structures of the epidermis which have numerous physical and physiological functions in various developmental stages of the plant’s life cycle [1,2]. Particularly trichomes with glandular activity have attracted scientific interest due to their ability to produce and spread a multitude of highly specialized metabolites for organismic interaction and their suitability for human use [3]. Glandular trichomes occur in nearly all plant families with a broad variety of different morphological types [2], which in some cases such as in Lamiaceae, Solanaceae or Asteraceae, have gained model character for investigation of developmental and biosynthetic processes [4,5,6].

Sunflower and many closely related species of the Helianthinae are known to possess at least two types of multicellular glandular trichomes: the biseriate capitate glandular trichomes (CGTs) and the uniseriate linear glandular trichomes (LGTs) [7]. Both types occur on the leaf blade of the common sunflower *Helianthus annuus* L., but LGTs are also located on petioles, stems and flower parts, with the highest density on the abaxial surface of phyllaries [8]. The formation of CGTs and LGTs starts in the earliest phase of organ development [9,10]. The production of bioactive sesquiterpene lactones (STLs) has directed the scientific interest primarily on CGTs, and numerous investigations on their occurrence [11], cytology [9,12], biosynthetic activity [13] and function [14,15] have been conducted. To the contrary, relatively few reports have focused on the LGTs of sunflower which are known to produce bisabolene-type sesquiterpenes (SLs) [16] as well as polymethoxylated flavones [17,18]. The localization of LGTs on different plant parts, their morphology and biosynthetic activity, in particular trichome cells, have been described [8], but the investigation of characteristic ultrastructural features of the different trichome cells is still missing. The data of this report will present details on the cytological development of LGTs from the pre-biosynthetic to the senescent stage using light and fluorescence microscopy (LM/FM) as well as transmission electron microscopy (TEM).

## 2. Results

**Figure 1:**
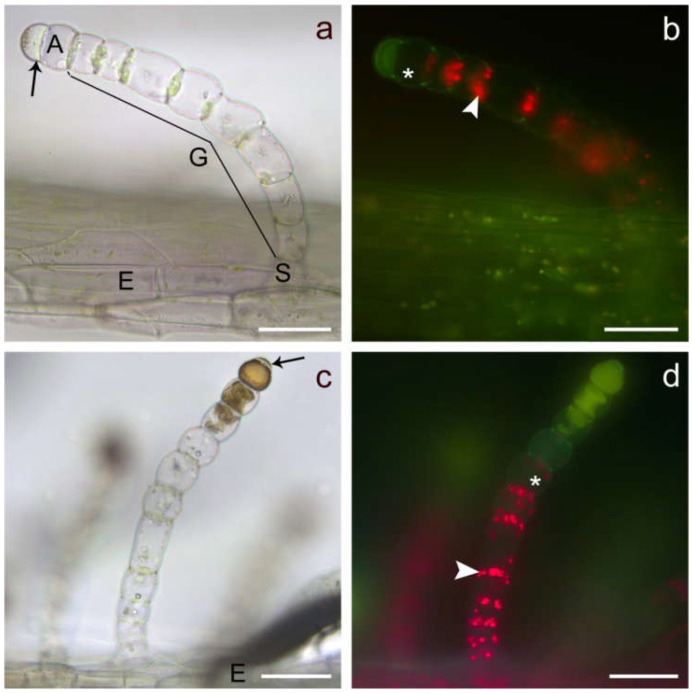
Light (**a**,**c**) and fluorescence (**b**,**d**) microscopic images of fully developed linear glandular trichomes from the stem epidermis in the early secretory stage (**a**,**b**) and from leave veins (**c**,**d**) in an advanced secretory stage. The trichomes consisted of a basal cell (obscured here) anchored in the epidermis (E), followed by a stalk cell (S) and 6–10 barrel-shaped glandular cells (G) with large vacuoles and plastids (white arrowheads). A mostly rounded apical cell (A) which could show irregular protuberances of subcuticular spaces (black arrows) formed the tip of the trichome. Cell which have lost vitality are marked by a star. Scale bar = 50 µm.

**Figure 2:**
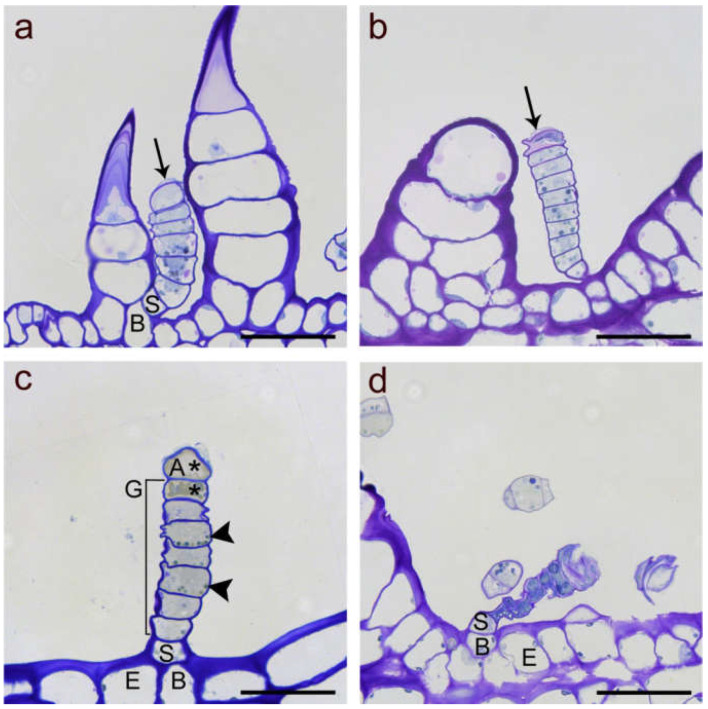
Light microscope images of linear glandular trichomes from phyllaries (**a**,**d**) and leave veins (**b**,**c**) in different developmental stages stained with Toluidine Blue O. (**a**) Early stage of a biosynthetically active LGT (between two nonglandular trichomes) showing strong vacuolization in the basal (B) and stalk cells (S), followed by five glandular cells rich in plasma and organelles and the apical cell starting with formation of a subcuticular cavity (black arrow). (**b**) Advanced stage of a biosynthetic activity LGT as indicated by progressed separation of the cuticle and extracellular storage of metabolites at the tip (black arrow). The apical cell had started degeneration and the wall partly collapsed. (**c**) Deposition of metabolites and cell degeneration (marked by stars) in the apical cell (A) and the adjacent glandular cell (G) while lower glandular cells still showed intact plastids (black arrowheads). The stalk cell showed thick lateral cell walls. (**d**) Senescent, degenerated trichome of the post-secretory stage with collapsed glandular cells but still vital stalk (S) and basal (B) cell in the epidermis (E). Scale bar = 50 µm.

LGTs located on stems (Figure 1a,b), leaf blades (Figure 1c,d), petioles or phyllaries (involucral bracts) showed very similar architecture. This accounted for both investigated genotypes. The sunflower line HA 300 and garden cultivar Giganteus LGTs consisted of four structurally different types of cells (Figure 2) starting with a basal cell as an anchor in the epidermis, followed by a single thick-walled stalk cell, a variable number of barrel-shaped glandular cells and ending with a dome-like apical cell, sometimes baring a small subcuticular space (Figure 1 and Figure 2). The number of glandular cells on LGTs of leaves and stem sections in the lower half of the plant ranged between five and seven, whereas LGTs on the upper half of the plant and phyllaries mostly showed between six and eight glandular cells. LGTs located at a certain plant part showed the identical developmental stage (pre-secretory, secretory or post-secretory stage, depending on the age of the investigated organ), thus confirming the previous report [10] of their simultaneous formation in the early stage of organ development.

Fluorescence microscopy of mature trichomes in the secretory stage revealed the presence of chlorophyll in the vital glandular cells (Figure 1b,d, white arrowheads) and the deposition of yellowish-green fluorescent metabolites which started in the apical cell and in the subcuticular space. In trichomes of advanced secretory stage, the deposition of compounds progressed downwards, simultaneously proceeding with the degeneration of glandular cells as indicated by the loss of chlorophyll and cellular compartmentation (Figure 1c,d).

Microscopic analysis of LGTs in different biosynthetically active stages confirmed the successive loss of cellular vitality from the apical cell to the adjacent glandular cell and further downwards in the course of trichome aging (Figure 2). Usually, the deposition of metabolites ended when the uppermost three or four glandular cells had lost vitality. Trichomes of this stage, when observed under a dissecting microscope, appeared bicolored with a clear, whitish base and an amber-colored front part (data not shown).

**Figure 3:**
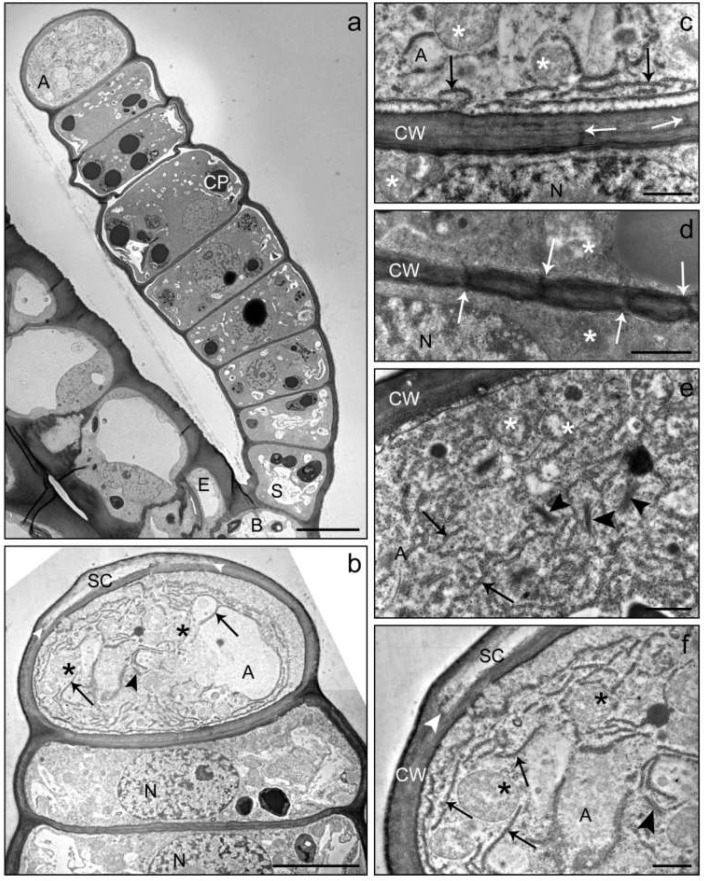
TEM images of a linear glandular trichome at the transition from the pre-secretory (**a**) to the secretory (**b**) stage. The overview (**a**) shows a young fully developed trichome anchored in the epidermis (EC) of an involucral bract at the sunflower capitulum. The trichome consisted of a basal cell (B), a stalk cell (S), 7 glandular cells and the apical cell (A). Vacuolization had just started in the basal and stalk cells, the apical cell did not yet show structures of compound accumulation or organ degradation and had not yet developed a subcuticular cavity. Chloroplasts (CP) in the glandular cells showed large electron-dense inclusions. (**c**) Cell wall between the apical cell (A) and uppermost glandular cell with plasmodesmata (white arrows). The apical cell contained many mitochondria (stars) and an extended rough endoplasmic reticulum (black arrows) close to the cell wall (CW). In the adjacent glandular cell, the nucleus (N) was visible. (**d**) Cell wall (CW) between two glandular cells from the middle of the trichome showing densely located plasmodesmata (white arrows) and mitochondria (stars). (**b**,**e**,**f**) Apical cell and glandular cells at the early secretory stage of development. The apical cell showed numerous mitochondria (stars), large amounts of rough endoplasmic reticulum (black arrows) and Golgi vesicles (black arrowheads). The cuticle started to separate (white arrowhead) from the cell wall to form a subcuticular cavity (SC). Scale bar: 10 µm (**a**)/5 µm (**b**)/1 µm (**c**–**f**).

**Figure 4:**
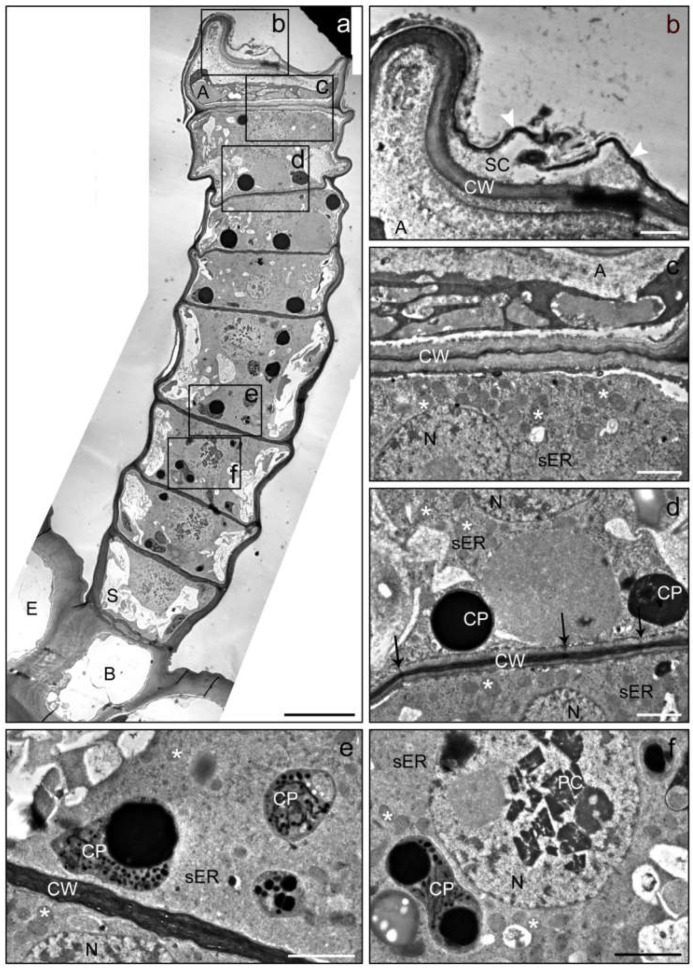
TEM images of a linear glandular trichome from a leaf vein in a late secretory stage. Frames in the overview (**a**) mark positions of the details (**b**–**f**). (**b**) The apical cell (A) showed strong degeneration and was partly collapsed; the subcuticular cacity (SC) between the cell wall (CW) and the cuticle (white arrowheads) seemed to have collapsed through rupture of the cuticle. (**c**) The cytosol of the apical cell (A) lacked clearly defined organelles, whereas the adjacent glandular cell was still vital and contained an intact nucleus (N), numerous mitochondria (stars) and smooth (sER) endoplasmic reticulum. (**d**,**e**) Glandular cells in the upper and central part of the trichome showing plastids (CP) with electron-dense inclusions and plasmodesmata (black arrows) in the periclinal cell wall (CW). (**f**) Glandular cell in the lower trichome region baring crystals (PC; presumably of proteins) in the nucleus. The number and volume of vacuoles increased towards the trichome base. Scale bar: 10 µm (**a**)/1 µm (**b**–**f**); (E) epidermis cell, (B) basal cell, (S) stalk cell.

**Figure 5:**
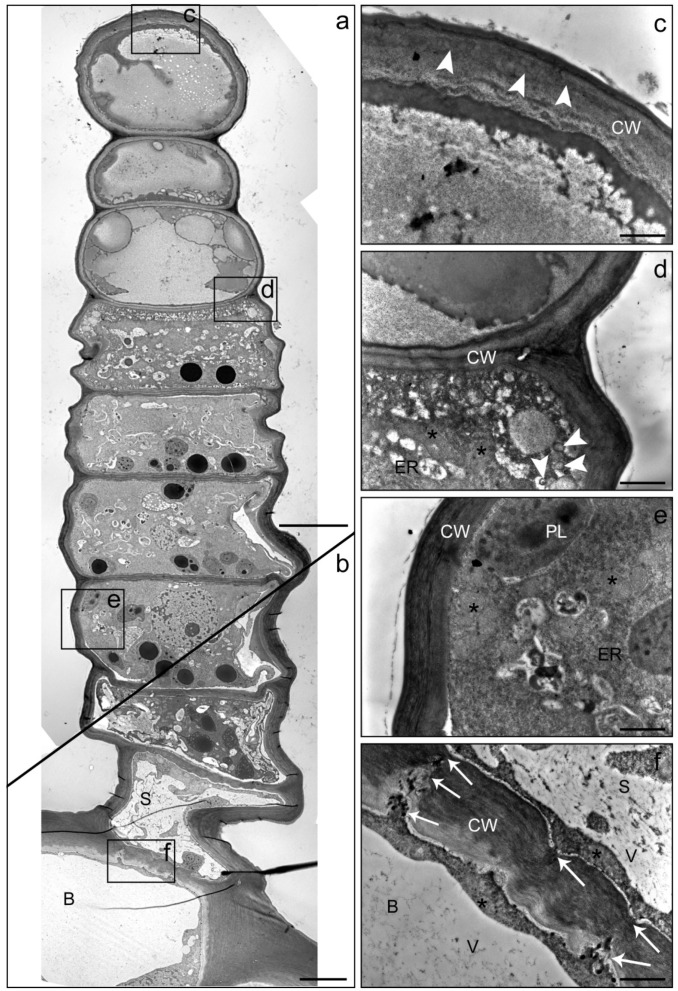
TEM images of a linear glandular trichome from a leaf vein in a late secretory stage. Frames in the photomerged overview (**a**,**b**) mark positions of the details (**c**–**f**). (**a**) The three uppermost cells were filled with metabolites, the cellular compartmentation had collapsed and organelles had disintegrated. (**c**) The cell wall (CW) of the apical cell showed signs of loosening (white arrowheads), possibly indicating the formation of a subcuticular cavity. (**d**) The process of cellular degeneration progressed downwards from the third to the fourth cell (in which mitochondria (stars) and endoplasmic reticulum (ER) were still visible along with large amounts small vacuoles and vesicles (white arrowheads)). (**e**) Vital cells in the lower part of the trichome showed intact plastids (PL), mitochondria (stars) and endoplasmic reticulum (ER). (**f**) The stalk cell (S) and the basal cell (B) were characterized by large vacuoles (V) and numerous plasmodesmata (white arrows) in the thickened cell wall (CW). Scale bar: 10 µm (**a**,**b**)/1 µm (**c**–**f**).

Electron microscopy revealed ultrastructural details of the different cell types and cytological alterations during the maturation and senescence of trichomes (Figure 3, Figure 4 and Figure 5). LGTs at the beginning of biosynthetic activity consisted of a basal cell similar in structure as the surrounding epidermal cells containing a large vacuole and organelles in the thin plasma area close to the cell wall (Figure 3a). A single stalk cell with several vacuoles and normal chloroplasts followed and led over to a series of between five and eight glandular cells. These cells were rich in cytoplasm. They contained a prominent nucleus (Figure 3b), numerous mitochondria (Figure 3f) and enlarged plastids with electron-dense inclusions (Figure 3a). The cytoplasm showed extended layers of endoplasmic reticulum (Figure 3e). At the beginning of the secretory stage, vacuoles were small and located in the peripheral parts of the cytoplasm. The apical cell had no or very few and small plastids and no vacuoles (Figure 3b,f). Extended layers of endoplasmic reticulum and numerous Golgi vesicles were located in the peripheral parts of the cytoplasm (Figure 3f). In the outer cell wall, separation of the cuticle could be observed occasionally, indicating the formation of a subcuticular cavity and the start of secreting activity. This process may start at the very tip of the cell or at several lateral sites. The subcuticular cavity was mostly a small and flattened, lens-shaped space. The cell walls between the apical cell and the glandular cells contained numerous plasmodesmata (Figure 3c,d).

In an advanced secretory stage (Figure 4), the apical cell started to lose its vitality and organelles disintegrated (Figure 4b), whereas the adjacent glandular cell appeared still vital and contained intact organelles such as a large nucleus and mitochondria (Figure 4c). Glandular cells often showed electron-dense inclusions in the plastids (Figure 4d,e) and possessed numerous plasmodesmata in the periclinal cell wall. In glandular cells of the lower trichome region, crystals were found in the nucleus (Figure 4f). The number and volume of vacuoles increased towards the trichome base.

The process of cell degeneration gradually progressed downwards in the glandular cells with the age of the trichome and towards the end of the secretory phase may have comprised the upper two or three glandular cells (Figure 5). The intracellular deposition of secretory products became visible when cellular compartmentation started to disintegrate (Figure 5a,d). It was observed that the apical cells of LGTs often collapsed (Figure 4b) during experimental fixation, when the deposition of products had not been completed yet, whereas their form stayed stable when adjacent glandular cells had also incorporated secretory metabolites (Figure 5a,c). Even in the late secretory phase, glandular cells in the lower part of the trichome stayed vital and showed intact plastids, mitochondria and endoplasmic reticulum (Figure 5e). At this stage, the stalk cell was characterized by large vacuoles and numerous plasmodesmata in the thickened cell wall (Figure 5f).

## 3. Discussion

Linear trichomes similar to the LGTs of sunflower have been observed in all taxa of the genus *Helianthus* and also in many species of other genera of the tribus Heliantheae [8]. Despite this broad occurrence and the investigation of the metabolic profile of the glandular products [16,18], cytological and subcellular details of LGT have not been investigated up to date.

Uniseriate glandular trichomes usually show differentiation between cells along the hair [2] and a gradual transition between three distinct developmental stages from a pre-secretory to secretory and post-secretory phase [19]. This similarly accounts for the LGTs of sunflower in this study, where four morphologically different cell types (basal, stalk, glandular and apical cell) along the trichome axis were identified. The general architecture of the LGTs on different sunflower organs was uniform, although the number of glandular cells was not found to be strictly defined or depending on trichome location. Contrary to previous studies, the investigation of different developmental stages now revealed that in the early phase of glandular activity, a slight separation of the cuticle at the apical cell takes place which may form lens-shaped subcuticular cavities. Interestingly, this was observed more frequently in trichomes of leaves located closer to the flower head than in leaves of the early plant development (data not shown). The subcuticular cavities are capable of taking up glandular secretions of yet uninvestigated chemical constitution (possibly essential oils, as assumed from previous GC/MS headspace experiments [18]). Unlike in sunflower CGTs, where the cuticle forms a globe-like compartment for taking up and storing the whole resinous exudates [20], the cuticle space in LGTs appeared transient and easily collapsed during sample preparation for microscopy. Due to the fragile constitution, subcuticular cavities were not observed in advanced developmental stages of LGTs. Whether rupture of the cuticle is necessary to release secreted compounds is unknown and requires further studies. However, alternative modes such as micro-channels [3] or minute pores in the intact cuticle for the release of volatile metabolites [21] are thinkable as well for the release of volatile compounds.

The functional peculiarity of the apical cell was also visible in its subcellular organization. In the pre-secretory stage, it was very rich in plasma, but contained no or only few plastids compared to the adjacent glandular cells. The high amount of endoplasmic reticulum and Golgi vesicles (Figure 3) is described as typical for a secretory cell [3]. The compounds appeared to be predominantly produced by glandular cells beneath the apical cell. The high number of plasmodesmata in the cell wall between the apical and the adjacent glandular cells indicates the capacity for extensive metabolite exchange [22].

Among the glandular cells, only the upper ones (usually between two and four) appeared to participate in the biosynthesis and storage of the metabolites. The accumulation of fluorescent compounds suggests the presence of flavonoids [18], whereas the sesquiterpenes found in LGTs [16] seem to contribute more to the brownish depositions visible in older cells after losing activity. Such co-occurrence of flavonoid aglycones with terpenes is typical for glandular trichomes with lipophilic secretions [1]. The high number of plastids paired with extended layers of endoplasmic reticulum is an additional sign for high metabolic activity of these cells. This has been described as a common feature of glandular cells producing hydrophobic material (e.g., essential oils and nonvolatile terpenes) [23]. The electron-dense material in form of plastoglobules eventually consist of lipids and suggests biosynthetic participation of the plastids as similarly described for Fabaceae producing terpenoids in their trichomes [24].

The ultrastructure of the stalk and basal cell indicated a function different from the synthesis or secretion of plant metabolites. Large vacuoles and thick cell walls suggest a physical function for stabilizing and anchoring the LGT in the epidermis.

The cytological details shown in this study underlined the specialization of different cell types operating together in the complex structure of LGTs in sunflower. Moreover, the ultrastructure of the cells undergoes dramatic cytological reorganization in the course of trichome development with consequences for the metabolic activity and function. These observations should help plan future experiments for the investigation of metabolic pathways in trichomes [25,26,27].

## 4. Materials and Methods

### 4.1. Plant Material and Cultivation

Plants of *Helianthus annuus* L. (line HA300 and garden cultivar Giganteus) were used in this study. The plants were grown from seeds and cultivated for 4 weeks in a climate chamber with a photoperiod of 14-h light and a constant temperature of 21 °C. Subsequently, the plants were transferred to the field and cultivated outdoors until flowering. Numerous samples of stems, leaf blades and phyllaries from different plants of the two genotypes were collected from sunflowers in various stages of buds, closed inflorescences and in full flower.

### 4.2. Sample Preparation and Microscopy

Plant tissue with LGTs was fixed in 5% (*v*/*v*) glutaraldehyde buffered with 0.1 M sodium phosphate buffer (pH 7.2) for 1 h at room temperature. As previously described in [28], after three washing steps in buffer, a second fixation was carried out in 1% osmiumtetroxide buffered with 0.1 sodium phosphate buffer (pH 7.2). Three washing steps in buffer followed. The progressive lowering of temperature method was applied for dehydration (1.5 h in 30% ethanol at 4 °C; 1.5 h in 50% ethanol at 0 °C; overnight in 70% ethanol at −20 °C; 1.5 h in 100% ethanol at −20 °C). After warming to RT, the samples were embedded in LR-White Resin (Science Service, Munich, Germany). The infiltrated samples were transferred to gelatine capsules (Science Service, Munich, Germany) and polymerized at 60 °C for 36 h. For light microscopy, semi-thin cross sections (1500 nm) of the samples were produced using an ultratom (Ultracut UCT, Leica, Wetzlar, Germany) with glass knives. The sections were stained for 45 min in 0.05% aqueous Toluidine Blue O (Merck, Darmstadt, Germany). For transmission electron microscopy, ultrathin sections were prepared using a diamond knife (Drukker International; Cuijk, The Netherlands). The sections were collected on Pioloform-carbon-coated copper grids, stained with lead citrate (according to [29]) for 30 s followed by uranyl acetate (1%) for 10 min and lead citrate for 30 s, and investigated in a transmission electron microscope (EM 10, Zeiss, Oberkochen, Germany) at 60 kV. Fresh trichomes and semi-thin sections were investigated with light and fluorescence microscopes Axioplan (Zeiss, Göttingen, Germany) coupled to a digital camera (Leica DCM 2900, Wetzlar, D). Chlorophyll distribution of untreated LGTs was observed under UV light with Filter 5395 nm/440 nm (Zeiss, Göttingen, Germany). Negatives of EM images were scanned (Epson Perfection 2450 Photo; Meerbusch, Germany). Brightness and contrast of all images were adjusted using Photoshop CS2 (Adobe; Berlin, Germany). The staple technique “photomerge” in the Photoshop program was used to prepare overview images of the ultrathin sections.

## Data Availability

The original data are provided in the manuscript.

## References

[1] Wagner G.J., Wang E., Shepherd R.W. (2004). New approaches for studying and exploiting an old protuberance, the plant trichome. Ann. Bot..

[2] Werker E. (2000). Trichome diversity and development. Adv. Bot. Res..

[3] Muravnik L.E., Ramawat K.G., Ekiert H.M., Goyal S. (2020). The structural peculiarities of the leaf glandular trichomes: A review. Plant Cell and Tissue Differentiation and Secondary Metabolites, Reference Series in Phytochemistry.

[4] Dai X., Wang G., Yang D.S., Tang Y., Broun P., Marks M.D., Sumner L.W., Dixon R.A., Zhao P.X. (2010). TrichOME: A comparative omics database for plant trichomes. Plant Physiol..

[5] Croteau R.B., Davis E.M., Ringer K.L., Wildung M.R. (2005). (−)-Menthol biosynthesis and molecular genetics. Naturwissenschaften.

[6] Duke M.V., Paul R.N., Elsohly H.N., Sturtz G., Duke S.O. (1994). Localization of artemisinin and artemisitene in foliar tissues of glanded and glandless biotypes of *Artemisia annua*. Int. J. Plant Sci..

[7] Spring O., Bienert U., Klemt V. (1987). Sesquiterpene lactones in glandular trichomes of sunflower leaves. J. Plant Physiol..

[8] Aschenbrenner A.K., Horakh S., Spring O. (2013). Linear glandular trichomes of *Helianthus* (Asteraceae): Morphology, localization, metabolite activity and occurrence. AoB Plants.

[9] Göpfert J.C., Heil N., Conrad J., Spring O. (2005). Cytological development and sesquiterpene lactone secretion in capitate glandular trichomes of sunflower. Plant Biol..

[10] Aschenbrenner A.-K., Amrehn E., Bechtel L., Spring O. (2015). Trichome differentiation on leaf primordia of *Helianthus annuus* (Asteraceae): Morphology, gene expression and metabolite profile. Planta.

[11] Spring O. (2000). Chemotaxonomy based on metabolites from glandular trichomes. Adv. Bot. Res..

[12] Amrehn E., Heller A., Spring O. (2014). Capitate glandular trichomes of *Helianthus annuus* (Asteraceae): Ultrastructure and cytological development. Protoplasma.

[13] Göpfert J.C., MacNevin G., Ro D.K., Spring O. (2009). Identification, functional characterization and developmental regulation of sesquiterpene synthases from sunflower capitate glandular trichomes. BMC Plant Biol..

[14] Spring O., Kupka J., Maier B., Hager A. (1982). Biological activities of sesquiterpene lactones from *Helianthus annuus*: Antimicrobial and cytotoxic properties; influence on DNA, RNA, and protein synthesis. Z. Naturforsch. Sect. C.

[15] Prasifka J.R., Spring O., Conrad J., Cook L.W., Palmquist D.E., Foley M.E. (2015). Sesquiterpene lactone composition of wild and cultivated sunflowers and biological activity against an insect pest. J. Agric. Food Chem..

[16] Spring O., Rodon U., Macias F.A. (1992). Sesquiterpenes from non-capitate glandular trichomes of *Helianthus annuus* L.. Phytochemistry.

[17] Brentan Silva D., Aschenbrenner A.K., Lopes N.P., Spring O. (2017). Direct analyses of secondary metabolites by mass spectrometry imaging (MSI) from sunflower (*Helianthus annuus* L.) trichomes. Molecules.

[18] Spring O., Pfannstiel J., Klaiber I., Conrad J., Beifuß U., Apel L., Aschenbrenner A.-K., Zipper R. (2015). The nonvolatile metabolome of sunflower linear glandular trichomes. Phytochemistry.

[19] Ascensao L., Pais M.S. (1998). The leaf capitate trichomes of *Leonotis leonurus*: Histochemistry, ultrastructure and secretion. Ann. Bot..

[20] Spring O. (1989). Microsampling: An alternative approach using sesquiterpene lactones for systematics. Biochem. Syst. Ecol..

[21] Giuliani C., Ascrizzi R., Corrà S., Maleci B.L., Flamini G., Fico G. (2017). *Salvia uliginosa* Benth: Glandular trichomes as bio-factories of volatiles and essential oil. Flora.

[22] Waigmann E., Zambryski P. (2000). Trichome plasmodesmata: A model system for cell-to-cell movement. Adv. Bot. Res..

[23] Lange B.M., Turner G.W. (2013). Terpenoid biosynthesis in trichomes—Current status and future opportunities. Plant Biotechnol. J..

[24] De Vargas W., Fortuna-Perez A.P., Lewis G.P., Piva T.C., Vatanparast M., Machado S.R. (2019). Ultrastructure and secretion of glandular trichomes in species of subtribe Cajaninae Benth (Leguminosae, Phaseoleae). Protoplasma.

[25] Tissier A. (2012). Glandular trichomes: What comes after expressed sequence tags?. Plant J..

[26] Huchelmann A., Boutry M., Hachez C. (2017). Plant glandular trichomes: Natural cell factories of high biotechnological interest. Plant Physiol..

[27] Frey M., Klaiber I., Conrad J., Spring O. (2020). CYP71BL9, the missing link in costunolide synthesis of sunflower. Phytochemistry.

[28] Amrehn E., Aschenbrenner A.-K., Heller A., Spring O. (2016). Localization of sesquiterpene lactone biosynthesis in cells of capitate glandular trichomes of *Helianthus annuus* (Asteraceae). Protoplasma.

[29] Reynolds E.S. (1963). The use of lead citrate at high pH as an electron-opaque stain in electron microscopy. J. Cell Biol..

